# What is that? HIV-negative plasmablastic lymphoma with intramuscular masses

**DOI:** 10.1259/bjrcr.20150211

**Published:** 2016-11-02

**Authors:** Salvador Alandete, Maria Dolores Monedero, M Angeles Meseguer, Fructuoso Delgado

**Affiliations:** Department of Radiology, Hospital Universitario Doctor Peset, Valencia, Spain

## Abstract

Plasmablastic lymphoma is a relatively new clinical entity described as a distinct subtype of diffuse large B-cell lymphoma, although in the last decade several case reports and series have been published. This case is presented because of its rarity, as this pathology is rare in immunocompetent patients and intramuscular masses are present. We report the case of a 63-year-old male with no significant clinical background. He was referred to the emergency department of our hospital with a 10-day history of pain on the left side of the chest that was described as burning and spreading to the right side. On physical examination, he had no fever or recent weight loss. The abdomen was soft and non-distended, and no peritoneal signs were present but he had three palpable masses located in the soft tissues of the breast, right gluteal region and left leg. Histological examination of the biopsy specimens disclosed the diagnosis of plasmablastic lymphoma. To our knowledge, this will be the second case report referring to intramuscular masses in the English language literature.

## Clinical history

A 63-year-old male with no signiﬁcant medical history, was referred to the emergency department of our hospital with a 10-day history of pain on the left side of the chest that was described as burning and spreading to the right side.

On physical examination, he had no fever or recent weight loss. The abdomen was soft and non-distended, and no peritoneal signs were present but he had three palpable masses located in the soft tissues of the breast, right gluteal region and left leg.

**Figure 1.**
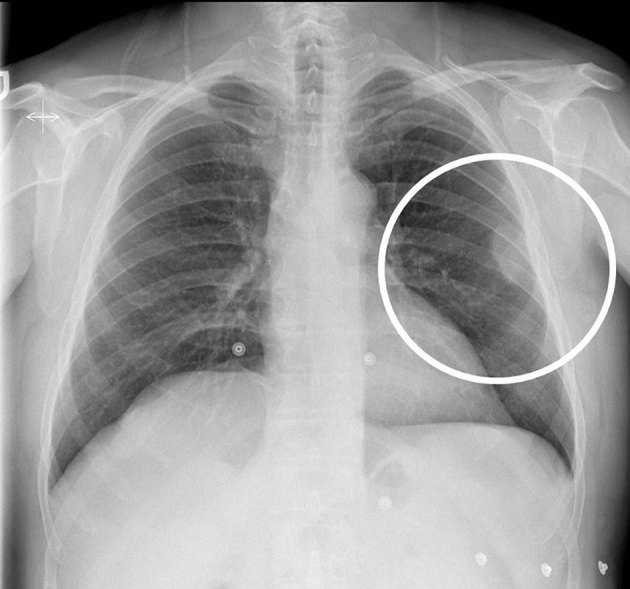
Chest X-ray showing a rounded subpleural mass in the upper left hemithorax (circle). No rib invasion is seen.

Blood analyses were normal and human immunodeficiency virus (HIV) and Epstein–Barr virus (EBV) tests were negative. Chest X-ray was performed (Figure 1) and showed a round subpleural mass in the upper left hemithorax without clear rib invasion.

**Figure 2.**
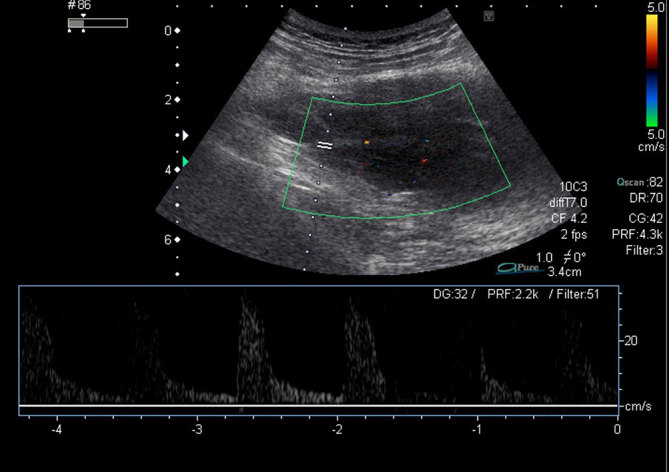
Doppler ultrasound showing a hypoechoic solid intramuscular mass affecting the right gluteus maximus and demonstrating arterial flow within the mass.

As he had three palpable masses, an ultrasound study was performed (Figure 2). Doppler ultrasound examination showed a hypoechoic solid intramuscular mass affecting the right gluteus maximus and demonstrating arterial flow within the mass.

**Figure 3.**
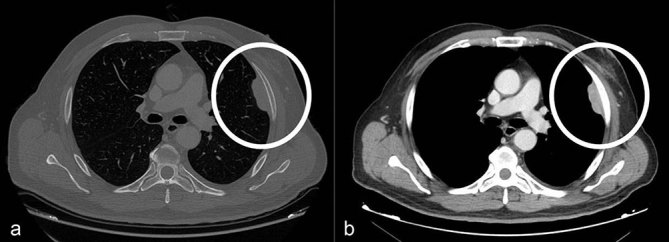
(a, b) CT scan of the thorax after i.v. contrast showing a solitary well-circumscribed, homogeneous solid subpleural mass (circles) in the upper left hemithorax with an obtuse angle between the mass and the chest wall. No rib invasion was noted.

The patient was admitted to the hospital in order to complete evaluation. During admission, a CT scan after i.v. contrast administration was performed to narrow the broad differential diagnosis. CT scan of the chest (Figure 3) confirmed the solitary well-circumscribed, homogeneous solid subpleural mass in the upper left hemithorax with an obtuse angle between the mass and the chest wall. No rib invasion was noted.

**Figure 4.**
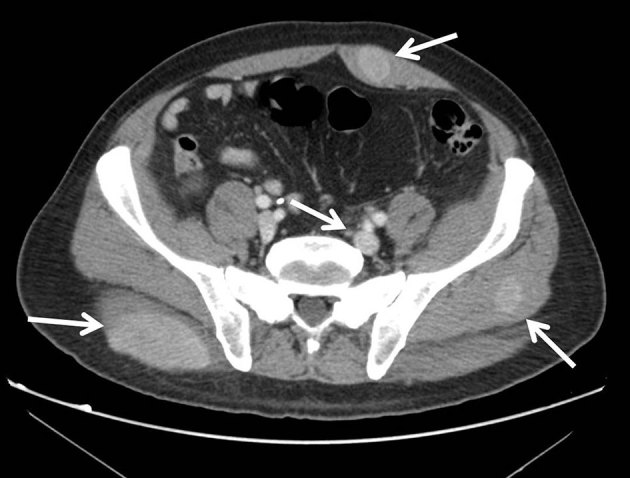
Upper abdominal CT scan after i.v. contrast showing multiple retroperitoneal masses afecting both adrenal glands, left kidney, retrocrural space, intraperitoneal and retroperitoneal fat (arrows).

**Figure 5.**
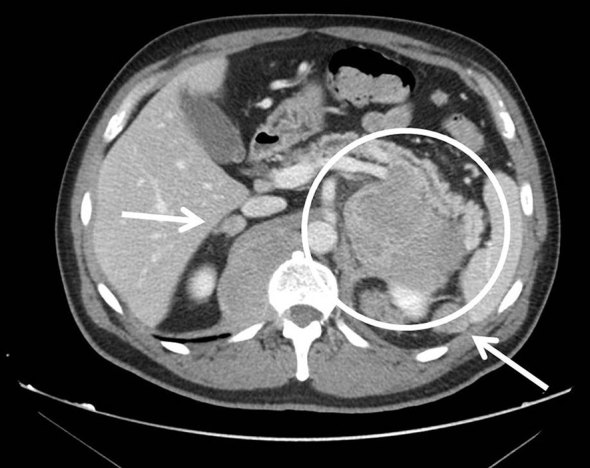
CT scan of the pelvis after i.v. contrast showing multiple intramuscular masses (circle).

Abdominopelvic CT scan (Figures 4 and 5) showed multiple retroperitoneal masses affecting both adrenal glands, left kidney, retrocrural space, intraperitoneal and retroperitoneal fat, and multiple intramuscular masses.

With all these imaging studies and clinical history, our differential diagnosis included metastases of unknown primary (melanoma and soft tissue sarcoma were considered) and lymphoproliferative disease.

Definitive diagnosis was made by biopsy that revealed high cellular density in the tumoral tissue with haematolymphoid cells, elevated mitotic index and high proliferative activity (Ki-67 expression > 90%). Immunohistochemistry was positive for vimentin, CD38, CD45, epithelial membrane antigen (EMA), immunohistochemical detection of multiple myeloma 1 (MUM1) and octamer transcription factor-2 (OCT-2). Epithelial, neuroendocrine (CD56), melanocytic, B-cell (CD20), T-cell and myeloperoxidase markers were negative. Although CD138 was negative, the inmunophenotype of these tumours is highly variable. These results confirmed the diagnosis of plasmablastic lymphoma (PBL).

Our patient was treated with a hyper-cyclophosphamide, vincristine, doxorubicin and dexamethasone (CVAD) chemotherapy protocol. Complete remission was achieved after four cycles and positron emission tomography-CT follow-up 8 months after the initial diagnosis showed complete remission.

The disease relapsed 1 year after the diagnosis and was treated with second-line chemotherapy cyclophosphamide, hydroxydaunorubicin, oncovin, prednisone (CHOP) protocol with partial response after two cycles. Also an autologous haematopoietic stem cell transplant was performed based on the poor prognosis of the disease. Currently, the patient is receiving chemotherapy without complete response.

## Discussion

PBL is a relatively new clinical entity that was first described in 1997^1^ as a distinct subtype of diffuse large B-cell lymphoma, which is characterized by its aggressive nature and plasmacytic differentiation. It is more commonly associated with HIV infection and has been estimated to comprise approximately 2% of all HIV-related lymphomas.

It is deﬁned by the World Health Organization^2^ as a “diﬀuse proliferation of large neoplastic cells most of which resemble B-cell immunoblasts, but in which all tumor cells have a plasma cell immunophenotype”. However, some investigators have suggested that PBL may represent an anaplastic subtype of plasmacytoma.^3^

In the past decade, several case reports and series have been published, accounting for no more than 590 cases based on the review by Castillo et al^4^ This case is presented because of its rarity. Our patient was HIV and EBV negative and intramuscular masses were present which is unusual.

Although the first cases of PBL were described in HIV-infected patients,^5^ an increasing number of reports are describing PBL being diagnosed in immunocompetent individuals. According to Castillo et al,^4^ PBL is more frequent in immunocompromised patients (HIV infected or transplant recipients; 72%) than in immunocompetent patients (28%).

Extranodal presentation was the most frequent (95% of patients), involving the oral cavity^6^ or the gastrointestinal tract.^7^ PBL in immunocompetent individuals seems to be more heterogeneous in terms of sites of involvement.^8,9^ Extraoral or intestinal sites have been reported, even though the appearance of muscle masses, as our case, is exceptional in the literature.^10^

Both immunocompromised and immunocompetent patients tend to present with late-stage disease. The typical clinical presentation in PBL cases is a male with a median age of 25–50 years with palpable lymph nodes and B symptoms that include weight loss, fever and night sweats. These symptoms are more commonly reported in HIV-negative patients compared with HIV-positive patients.^11^

Currently, very little is known about the molecular and genetic basis of PBL; however, there are several studies that attempt to clarify this aspect.^12^ Similar to other lymphomas, PBL has a strong association with EBV infection, but this was not a feature in our patient.

Despite progress in recent years, PBL remains a diagnostic challenge given its peculiar morphology and an immunohistochemical profile, which is very similar to plasma cell myeloma.^13^ The neoplastic cells are large with abundant cytoplasm and central oval vesicular nuclei with prominent nucleoli as noted in large immunoblasts. Its unique immunohistochemical profile may mislead pathologists, and potentially delay an accurate diagnosis and proper clinical treatment.^14^ PBL’s immunophenotype is similar to that of plasma cell neoplasms, positive for CD79a, interferon regulatory factor 4 (IRF-4)/MUM1 , BLIMP-1 , CD38 and CD138, and negative for B-cell markers CD19 and CD20.

Imaging findings were not specific and the main image-based diagnosis considered was metastases, but we found some clues that made us include lymphoproliferative disease in the differential diagnosis.

First, there were multiple focal lesions in a wide variety of locations,^15^ but without lungs, liver or bone infiltration, which are the typical sites of metastases. Also, the soft tissue mass located at the chest wall respected adjacent ribs, which was a remarkable finding in such an aggressive disease.^16^

Furthermore, some of the soft tissue masses in a retrocrural location resembled an “extramedullary haematopoiesis-like” distribution^17^ and that was one of the clues to include haematologic disease (in this particular case, lymphoma) as one of our differentials. Lymphomas are one of the well-known causes of enlarged retrocrural nodal masses.^18^

Definitive diagnosis was made by biopsy, although owing to everything mentioned above the diagnosis of lymphoma was suggested in our radiology report.

PBL also represents a therapeutic challenge, with early responses to therapy but with high relapse rates and poor prognosis.^19^ The common chemotherapy regimens that have been used with partial or complete response include CHOP and EPOCH, although treatment guidelines have not been established yet. Castillo et al’s^4^ recommendation for treatment of PBL is six cycles of infusional dose-adjusted EPOCH (with or without bortezomib) with intrathecal prophylaxis. Autologous transplant is considered optional in some cases.

An important aspect of the initial treatment of PBL is the use of chemotherapy, which has a high overall response rate of 77%. Patients with PBL who were not treated with chemotherapy invariably died, with a median survival of 3 months. In spite of the good response to chemotherapy, the median overall survival is 14 months, with a 5-year rate of 31%.^12^ Some of the variables associated with longer survival were early clinical stage, age > 60 years, advanced stage at diagnosis, bone marrow involvement and lack of treatment with chemotherapy

## Conclusions

Owing to its new clinical description and highly variable presentation, PBL represents a diagnostic challenge for radiologists and pathologists.

It is important to remember that multiple focal lesions in a wide variety of locations without a primary known malignant tumour, and with no infiltration of the lungs, liver or bone is an atypical feature of metastases, so radiologists should broaden their differential diagnosis and include lymphoma as a possibility, even when lymphadenopathies and splenomegaly are absent.

This case shows too that clinical presentation and imaging of lymphoma may resemble chest wall tumours, which is not a common feature. Understanding this rare subtype of B-cell lymphoma would benefit patient care by rendering early correct diagnosis, predicting clinical outcomes and initiating appropriate therapeutic strategies.

## Learning points

1. Although first cases of PBL were described in HIV-infected patients,^5^ an increasing number of reports are describing PBL being diagnosed in immunocompetent individuals.
2. Extranodal presentation was the most frequent (95% of patients), involving the oral cavity or the gastrointestinal tract but there are case reports of a wide range of locations being affected.
3. The appearance of intramuscular masses, as in our case, is exceptional in the literature.
4. Multiple focal lesions in a wide variety of locations, without lungs, liver or bone inﬁltration are not a common feature in metastasis. Lymphoma, and in this case, PBL, should be considered in the differential diagnosis.
5. Recognizing this rare subtype of B-cell lymphoma would beneﬁt patient care by rendering early correct diagnosis, predicting clinical outcomes and initiating appropriate therapeutic strategies.

## Consent

Written informed consent for the case to be published (including images, case history and data) was obtained from the patient for publication of this case report.
